# Advantage of magnifying narrow‐band imaging for the diagnosis of colorectal neoplasia associated with sessile serrated lesions

**DOI:** 10.1002/deo2.315

**Published:** 2023-12-01

**Authors:** Yuri Enomoto, Mitsuaki Ishioka, Akiko Chino, Hikari Kobayashi, Ryo Shimizu, Chihiro Yasue, Daisuke Ide, Masahiro Igarashi, Junko Fujisaki, Takahisa Matsuda, Yoshinori Igarashi, Shoichi Saito

**Affiliations:** ^1^ Department of Gastroenterology Cancer Institute Hospital of the Japanese Foundation for Cancer Research Tokyo Japan; ^2^ Department of Internal Medicine Division of Gastroenterology and Hepatology Toho University Omori Medical Center Tokyo Japan

**Keywords:** colorectal cancer, narrow‐band imaging, sessile serrated adenoma/polyp, sessile serrated lesion with dysplasia, sessile serrated lesion

## Abstract

**Objectives:**

This study aimed to extract endoscopic findings for diagnosing colorectal neoplasia associated with sessile serrated lesions (SSLs), which are of significant interest.

**Methods:**

To compare the magnifying narrow‐band imaging (NBI) findings with microscopic morphology, we classified SSLs into two groups: Group A SSLs included the majority of uniform SSLs and any dysplasia other than that classified as group B SSLs. Group B SSLs included SSLs with intramucosal and invasive carcinoma. We also quantitatively assessed visible vessels using ImageJ software.

**Results:**

This study included 47 patients with 50 group B SSLs who underwent endoscopic resection between 2012 and 2020. The results were retrospectively compared with those of 237 patients with 311 group A SSLs that underwent endoscopic resection. Using conventional white‐light endoscopy, significantly more group B SSLs had uneven shapes and some reddening compared to group A SSLs. The diagnostic odds ratios for group B SSLs were as follows: lesions with a diameter ≥10 mm, 9.76; uneven shape, 3.79; reddening, 15.46; and visible vessels with NBI, 11.32. Regarding visible vessels with NBI, the specificity and diagnostic accuracy for group B SSLs were 94.9% and 93.1%, respectively. The percentage of the vascular tonal area of NBI images was significantly larger for group B SSLs than for group A SSLs (3.97% vs. 0.29%; *p* < 0.01).

**Conclusions:**

SSLs with reddening and/or a diameter ≥10 mm are suspected to contain cancerous components. Moreover, visible vessels observed using magnifying NBI can serve as objective indicators for diagnosing SSLs with cancerous components with a high degree of accuracy.

## INTRODUCTION

The existence of the “serrated neoplastic pathway,” in which serrated lesions are the precursor lesions, has attracted attention.^1^, ^2^ In 2010, the World Health Organization (WHO) classified serrated colorectal lesions as hyperplastic polyps, traditional serrated adenomas, and sessile serrated adenoma/polyp (SSA/P)^3^; however, the inter‐rater agreement of their diagnosis has been reported to be low, even among expert pathologists.^4^ Additionally, there are some lesions with mixed features of each serrated lesion, thus causing confusion. In 2019, the 5th edition of the WHO classification used the term “sessile serrated lesion” (SSL) instead of SSA/P, and created a more comprehensive definition that indicates that serrated lesions with at least one crypt dilation are considered SSLs.^5^

The position regarding SSLs with dysplasia (SSLD) by the WHO classification differs in Japan and Western countries. In Japan, carcinoma is defined by nuclear atypia, cytoplasmic abnormalities, and abnormal glandular duct structures (irregular branching, meandering, fusion, etc.), irrespective of the invasive distance.^6^ Therefore, according to the WHO classification, SSLD encompasses what is termed “intramucosal carcinoma” in Japan. In contrast, in the West, carcinoma is defined as invasion beyond the mucosal muscularis mucosae into the submucosa^7^; hence, the notion of intramucosal carcinoma is absent. The 4th edition of the WHO classification proposed that SSA/P with cytological dysplasia are potentially malignant lesions, as did the 5th edition of the WHO classification, which also considered structural dysplasia as potentially malignant. However, the grading of dysplasia is not recommended, and its concept is inclusive of groups of lesions with different biological grades of malignancy.^5^, ^8^, ^9^

From a clinical perspective, the early diagnosis of precancerous lesions with high malignant potential is critical. We believe that it is feasible to distinguish cancerous components, including intramucosal carcinoma in Japan, from other types of dysplasia through endoscopic findings.^10^ This study aimed to evaluate endoscopic findings obtained with different modalities that are useful for diagnosing cancerous components of SSLs and objectively evaluating tumor blood vessels. Additionally, this study focused on the simplicity of magnified narrow‐band imaging (NBI).

## METHODS

### Target lesions and group definitions

In this study, the histopathological diagnosis of SSLs was made according to the 5th edition of the WHO classification criteria for SSLs.^5^ The Japanese Society for Cancer of the Colon and Rectum guidelines were used only for lesions with intramucosal adenocarcinoma in Japan.^6^

To avoid confusion caused by mixed pathological interpretations of SSLs in the West and Japan, SSLs were separated into group A and group B. Group A SSLs comprised the majority of uniform SSLs and any dysplasia other than that defined as group B SSLs. Group B SSLs comprised SSLs with intramucosal carcinoma according to the Japanese Society for Cancer of the Colon and Rectum criteria and invasive carcinoma with SSLs.

### Materials

This was a retrospective study. We enrolled 47 patients with 50 cases of group B SSLs that underwent endoscopic resection (including snare polypectomy, endoscopic mucosal resection, and endoscopic submucosal dissection) at our institute between January 2012 and December 2020. Because clinical encounters of group A SSLs were more frequent, we retrospectively extracted only those cases that had undergone endoscopic resection during the most recent year (January 2020 to December 2020) for comparison. Lesions resected by cold snare polypectomy were excluded. Thus, data from 237 patients with 311 cases of group A SSLs were extracted. All final histopathological diagnoses were made by two or more expert pathologists who were board‐certified by the Japanese Society of Pathology.

This study was approved by the ethics committee of the Institutional Review Board (no. 2020‐GA‐1091), and the requirement for written informed consent from patients was waived because of the retrospective nature of the study.

### Endoscopic findings required for endoscopic evaluation

**FIGURE 1:**
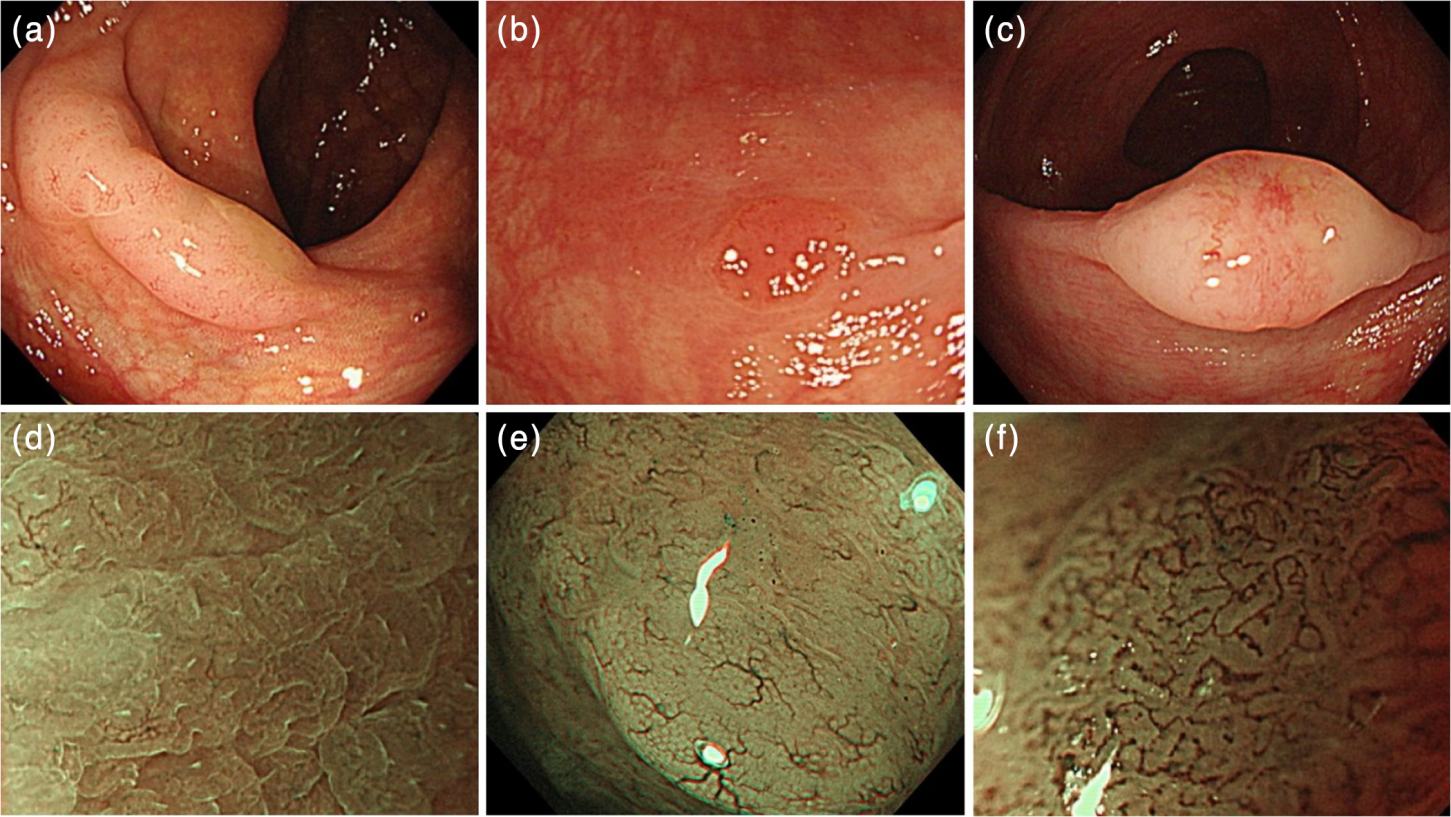
Representative endoscopic findings used to construct the evaluation criteria. (a) Uniform flat shape and uniformly translucent color tone. (b) Uneven shape with some reddening. (c) Uneven shape with some reddening. (d) Invisible vessel pattern on magnifying narrow‐band imaging (NBI). (e) Lacy vessel pattern (orderly network of thin‐caliber vessels) on magnifying NBI. (f) Visible vessel pattern on magnifying NBI.

All endoscopic images were obtained using a colonoscope with a magnification function (PCF‐Q260AZI or PCF‐H290ZI; Olympus). Typical endoscopic findings of group A SSLs and group B SSLs are shown in Figure 1. Endoscopic findings with white‐light conventional endoscopy were classified by the lesion shape (uniform flat or uneven shape, e.g., pedunculated morphology, double elevation, and central depression) and color tone (uniformly translucent or with some reddening). The NBI findings were classified by the vessel pattern (invisible or lacy vessel pattern [orderly network of thin‐caliber vessels] or visible vessels). Lesions were evaluated to determine the presence or absence of these endoscopic findings (Table 1). The endoscopic findings were evaluated by a single endoscopist (Yuri Enomoto). We used ImageJ software (National Institutes of Health) to confirm the usefulness of the vascular color density of NBI and ensure objectivity during the evaluation of visible blood vessels.^11^

**TABLE 1:** Characteristics of the patients and endoscopic findings of lesions.

			Group B SSLs (*n* = 50)	Group A SSLs (*n* = 311)	*p*‐value
Sex, male, N (%)			16 (32.0)	161 (51.8)	<0.01
Age, years, median, (IQR)			73 (67–78)	63 (52–70)	<0.01
Location, N (%)					0.82
Proximal colon			43 (86.0)	271 (87.1)	
Distal colon			7 (14.0)	40 (12.9)	
Size of the lesion, mm, median, (IQR)			20 (13–24)	12 (10–18)	<0.01
Endoscopic findings					
WLI	Shape	Uniform flat	33 (66.0)	290 (93.2)	<0.01
		Uneven shape	17 (34.0)	21 (6.8)	
	Color	Uniformly translucent	1 (2.0)	256 (82.3)	<0.01
		With some reddening	49 (98.0)	55 (17)	
NBI	Vessel	Invisible or lacy	6 (12.0)	285 (91.6)	<0.01
		Visible vessels	44 (88.0)	26 (8.4)	<0.01
	Surface	Expanded crypt openings	34 (68.0)	231 (74.3)	0.22

*Note*: Group A SSLs, uniformed SSLs and SSL with any dysplasia other than Group B SSLs; group B SSLs, SSL with intramucosal cancer component and invasive carcinoma; WLI, white light imaging; NBI, narrow‐band imaging.

### Image processing

**FIGURE 2:**
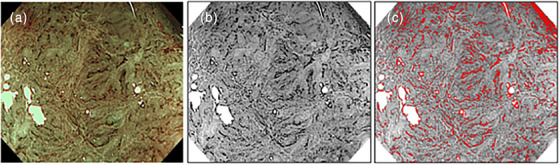
Quantification of neoplastic vessels with ImageJ software. (a) Original endoscopic image obtained with magnified narrow‐band imaging (80× magnification). (b) Green channel image decomposed by ImageJ software. (c) Extract of the color tone of blood vessels from the green channel image at a threshold of 70.

During image preprocessing, representative images of each case (80× magnification) loaded in ImageJ software were decomposed into three images using RGB color by selecting the “Image” > “Color” > “Split Channels” options. Then, the threshold was set to 70 to accurately divide the vascular area and image background by selecting “Image” > “Adjust” > “Threshold.” Finally, measurements of the areas of the extracted vascular tones were analyzed by selecting “Analyze” > “Measure” (Figure 2).

### Statistical analysis

Statistical analyses of the group A SSLs and group B SSLs were performed using the Mann–Whitney U test or chi‐square test. Univariate and multivariate analyses were performed to evaluate endoscopic factors predictive of group B SSLs. A univariate analysis was first performed with the presence or absence of carcinoma (including intramucosal carcinoma) with SSLs as the dependent variable and the endoscopic predictors (lesion location, lesion size, lesion shape, lesion color, and visibility of tumor vessels by NBI) as explanatory factors. Next, a multivariate analysis was performed using the forced entry method; only those predictors that showed statistical significance in the univariate analysis were included. All analyses were performed using IBM SPSS Statistics version 27 (IBM Corp.). Statistical significance was set at *p* < 0.05.

## RESULTS

### Characteristics of patients

Patient characteristics are shown in Table 1. Patients with group B SSLs were significantly older than those with group A SSLs (mean age, 73 years vs. 63 years; *p* < 0.01). Moreover, significantly more female patients had group B SSLs (84.0% [group B] vs. 48.2% [group A]; *p* < 0.01). Furthermore, group B SSLs were larger than group A SSLs (20 mm vs. 12 mm; *p* < 0.01). No significant difference in the lesion location of these two groups was observed.

### Endoscopic evaluation results

The results of the endoscopic evaluation are presented in Table 1. According to conventional white‐light endoscopy, significantly more group B SSLs were unevenly shaped (34.0% vs. 6.8%; *p* < 0.01) and had some reddening (98.0% vs. 17.7%; *p* < 0.01) compared to group A SSLs. Using magnifying NBI, the visible vessel rate was significantly higher for group B SSLs than for group A SSLs (88.0% vs. 8.4%; *p* < 0.01). The frequency of the endoscopic finding of expanded crypt openings on surface patterns using magnifying NBI was approximately 70% for group B SSLs and group A SSLs, with no significant difference between these groups.^12^

The diagnostic odds ratios of the endoscopic findings of group B SSLs were as follows: diameter ≥10 mm, 9.76 (95% confidence interval [CI]: 2.70–35.35); uneven shape, 3.79 (95% CI: 0.72–19.96); some reddening, 15.46 (95% CI: 1.28–186.19); and visible vessels, 11.32 (95% CI: 3.44–37.27; Table 2). When using NBI for group B SSLs, the sensitivity, specificity, positive predictive value (PPV), negative predictive value (NPV), and diagnostic accuracy of visible vessels were 82.0%, 94.9%, 87.2%, 97.0%, and 93.1%, respectively (Table 3).

**TABLE 2:** Univariate and multivariate analyses of the predictive endoscopic factors for colorectal neoplasia associated with sessile serrated lesions.

		Univariate		Multivariate	
Factors	Group B/A SSLs	RR (95% CI)	*p*‐value	RR (95% CI)	*p*‐value
Location					
Proximal colon	43/271	Ref			
Distal colon	7/40	1.10 (0.46–2.62)	0.82	–	–
Size of the lesion, mm					
< 10	4/118	Ref		Ref	
≥ 10	46/193	5.50 (2.12–14.26)	<0.01	9.76 (2.70–35.35)	< 0.01
WLI‐Shape					
Uniform flat (0‐IIa)	33/290	Ref		Ref	
Uneven shape	17/21	7.11 (3.42–14.82)	<0.01	3.79 (0.72–19.96)	0.12
WLI‐Color					
Uniformly translucent	1/256	Ref		Ref	
With some reddening	49/55	228.07 (30.83–1687.23)	<0.01	15.46 (1.28–186.19)	0.03
NBI‐Vessel					
Invisible, lacy	6/285			Ref	
Visible vessel	44/26	80.39 (31.31–206.35)	<0.01	11.32 (3.44–37.27)	< 0.01

*Note*: Group A sessile serrated lesions (SSLs): uniformed SSLs and SSL with any dysplasia other than Group B SSLs; group B SSLs, SSL with intramucosal cancer component and invasive carcinoma; WLI, white light imaging; NBI, narrow‐band imaging; ECO, expanded crypt openings; RR, risk ratio; 95% CI, 95% confidence interval.

**TABLE 3:** Diagnostic accuracy of endoscopic findings to group B sessile serrated lesions (SSLs).

	Sensitivity	Specificity	PPV	NPV	Accuracy
Size of the lesion, ≥ 10mm	92.0%	5.8%	13.6%	81.8%	17.7%
Uneven shape	34.0%	93.2%	44.7%	89.8%	85.0%
With some reddening	98.0%	82.3%	47.1%	99.6%	84.5%
Visible vessels on NBI	82.0%	94.9%	87.2%	97.0%	93.1%

*Note*: Group B SSLs, SSL with intramucosal cancer component and invasive carcinoma; PPV, positive predictive value; NPV, negative predictive value; NBI, narrow‐band imaging.

### Objective evaluation of visible vessels using NBI

**FIGURE 3:**
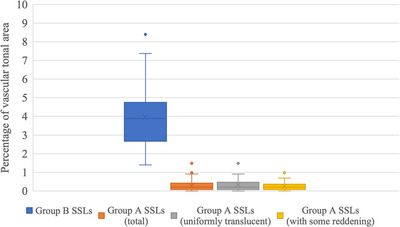
Percentage of blood vessel coloration calculated by the software. *p < 0.01.

ImageJ software was used to objectively evaluate visible vessels. The percentage of the vascular tonal area on the endoscopic images obtained by magnified NBI was significantly larger for group B SSLs than for group A SSLs (3.97% vs. 0.29%; *p* < 0.01). Furthermore, even when the group A SSLs were divided into two groups, with one comprising SSLs with only uniform translucency and one comprising SSLs with some reddening, which were considered difficult to distinguish from group B SSLs under white‐light imaging, the percentage of the vascular tonal area on the endoscopic images obtained by magnified NBI was significantly different (Figure 3).

### Treatment outcomes of invasive carcinoma with SSLs

Among 50 patients with group B SSLs treated with endoscopic resection, 84.0% (42/50) had no risk factors for lymph node metastasis; therefore, curative resection was considered. Of the remaining eight patients, four had deep submucosal invasion (≥1000 μm) and positive lymphovascular invasion, two had deep submucosal invasion alone, and two had only one risk factor (lymphovascular invasion). Three patients underwent additional colectomy; however, lymph node metastasis was not found. The other five patients were followed up annually with endoscopy and computed tomography imaging, and metastatic recurrence was not observed.

## DISCUSSION

In this study, we identified endoscopic findings that are useful for differentiating group B SSLs from other SSLs. Using white‐light imaging, lesions with a diameter ≥10 mm and those with an uneven shape often have cancerous components, including intramucosal carcinoma in Japan. Furthermore, if reddening is observed in part of the lesion, then adding magnifying NBI to identify visible vessels enables a highly accurate diagnosis of group B SSLs. To the best of our knowledge, this is the first study to quantitatively evaluate tumor blood vessels of group B SSLs using ImageJ software.

Among SSLD, precancerous lesions of microsatellite instability‐high carcinomas are considered a type of dysplasia by the 5th edition of the WHO classification. However, because SSLD is considered difficult to diagnose using morphological findings, clinical findings, such as patient sex, age, lesion location, and lesion size, have been suggested as indications for endoscopic resection.^13^

Recently, endoscopic resection techniques have become more diverse, and views regarding the indications for SSL treatment have been reported. Hattem et al. reported that piecemeal cold snare polypectomy for large SSLs (≥20 mm) could be a safe new standard treatment rather than conventional endoscopic mucosal resection.^14^ Conversely, we encountered a case of invasive carcinoma recurrence 5 years after endoscopic piecemeal mucosal resection for SSLs of the proximal colon in an elderly woman; therefore, we believe that it is crucial to determine lesions at high risk for being precancerous and perform en bloc complete resection and an accurate histopathological evaluation to prevent recurrence.^15^

It is important to be able to identify which lesions with histologic changes and SSLs are candidates for aggressive treatment (e.g., endoscopic submucosal dissection). Furthermore, it is equally crucial to differentiate the lesions that warrant prophylactic resection (cold snare polypectomy and piecemeal polypectomy) before treatment. Thus, a precise pretreatment diagnosis is critical to selecting the treatment and treatment modalities for SSLs.

To confidently contrast the findings of the magnified endoscopic images with the phenomena shown by microscopic morphology, we used the medical records of the pathologists at our institution and classified them into group A SSLs and group B SSLs before examining them.

According to previous studies, the following findings were useful for differentiating SSLD and carcinoma: lesion diameter ≥10 mm; uneven shape (pedunculated/semi‐pedunculated morphology, double elevation, and depression); and Japan NBI Expert Team classifications other than type 1.^16^, ^17^, ^18^ Group B SSLs in this study are not synonymous with SSLD reported previously. However, lesion diameter ≥10 mm was an independent predictor of group B SSLs in the present study, consistent with previous reports. Furthermore, the selection of lesions for treatment based on their size is considered effective. However, although the sensitivity was relatively good (92.0%), the PPV was low (13.6%), and the positive diagnosis rate was 17.7%. As with conventional adenomas, the frequency of carcinomatous lesions generally increases with the increasing SSL size; however, the rate is lower for SSLs than for conventional adenomas when the lesions are the same size.^19^, ^20^, ^21^ Additionally, in the present study, there were four cases of intramucosal carcinoma with SSLs <10 mm, suggesting the need for more detailed differentiation using factors other than the lesion size.

When some reddening was observed in a part of the lesion, the sensitivity for group B SSLs was as high as 98.0% (Table 3), and it appeared to be an endoscopic finding that is useful for identifying lesions with cancerous components; however, the PPV was not high (47.1%). The reason for the low PPV was that even noncancerous lesions can be accompanied by reddening (Figure S1). We considered this reddening to be an endoscopic finding associated with inflammation and/or congestion; however, it could not be proven histopathologically because this was a retrospective study. Although it is difficult to distinguish between group A SSLs and group B SSLs using white‐light imaging alone, the addition of NBI enables the diagnosis to be determined with high accuracy (93.1%) because the reddening of group B SSLs is caused by the dilated vessel diameter and increased vessel density.

**FIGURE 4:**
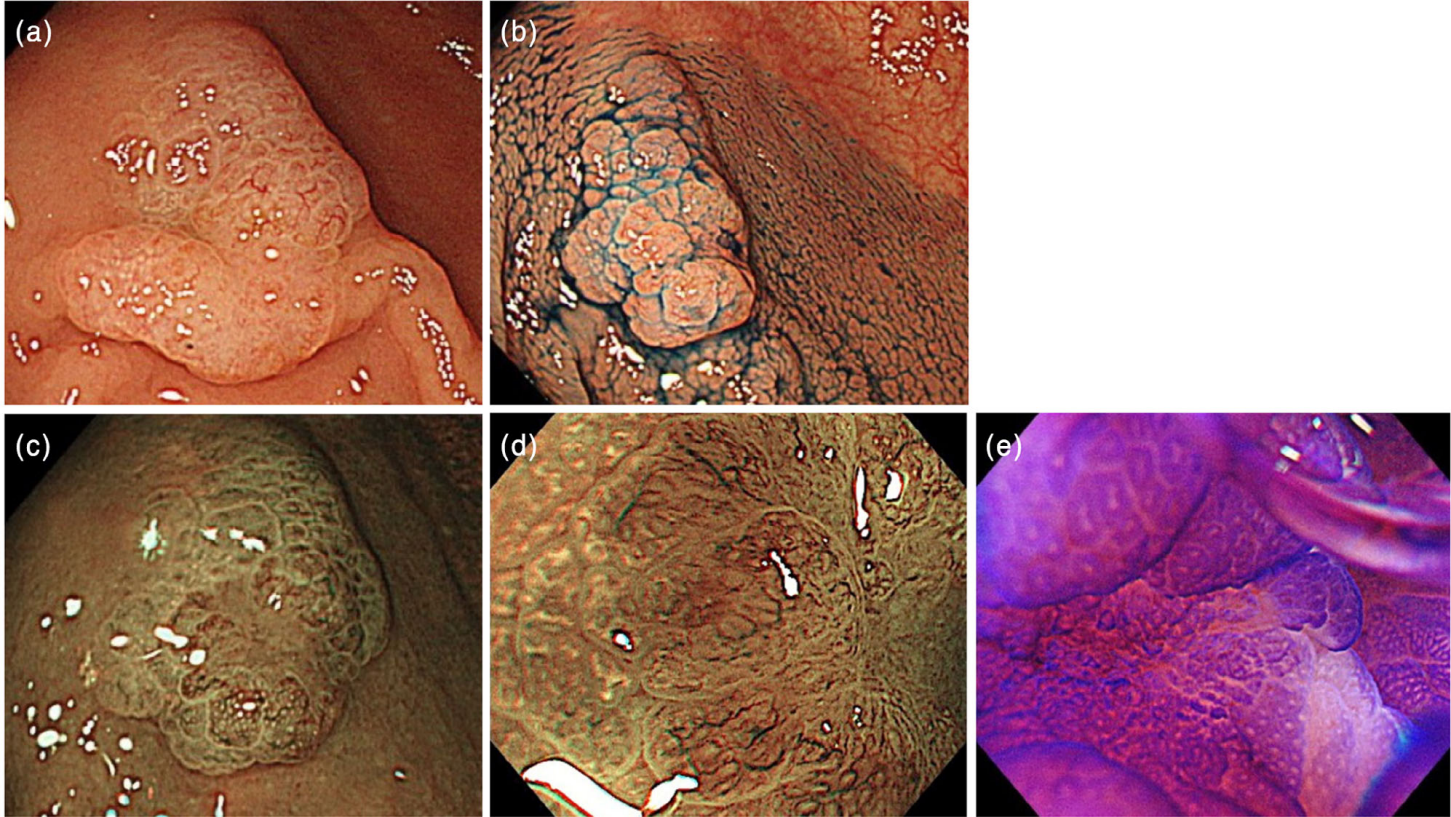
The process of diagnosing sessile serrated lesions (SSLs) with cancerous components in actual clinical practice. (a and b) Lesions with a diameter ≥10 mm and/or some reddening are detected. (c and d) Visible vessels observed using magnifying narrow‐band imaging (NBI) can be an objective indicator of the diagnosis of SSLs with cancerous components. (e) Type Vi pit pattern observed using crystal violet staining in the same area where visible vessels are recognized by magnifying NBI and diagnosed as cancerous components.

The density of visible vessels is believed to reflect the difference in malignancy of group A SSLs and group B SSLs. The ImageJ software results suggested that high‐risk lesions may be efficiently extracted from a large number of SSLs by using the color tone of NBI images. In actual clinical practice, after discovering high‐risk lesions using NBI, a more accurate preoperative diagnosis, including the diagnosis of the type V pit pattern, using magnifying endoscopy with crystal violet staining (Figure 4) and complete contrast with postoperative pathology specimens should be prospectively determined.^11^

This study had several limitations. First, this was a single‐center, retrospective study. Because group B SSLs are rare, we could not obtain a sufficient number of cases to validate the results. Furthermore, the duration of the case collection period was extensive to allow a greater number of group B SSLs to be acquired; however, this posed challenges when attempting to thoroughly evaluate the images of all group A SSL cases within the same timeframe. Because of the substantial volume of cases involved, only the most recent SSL cases within a 1‐year timeframe were incorporated in this study, which could have introduced bias. Second, unlike pit pattern diagnosis, it is difficult to contrast morphologically the vascular pattern using NBI with a pathological specimen obtained after resection. Third, image analysis using ImageJ software in this study was not intended to be used for the real‐time diagnosis; instead, it was performed as a quantitative evaluation method to confirm the objectivity of the visible vessels observed in SSLs with carcinoma (group B SSLs). Fourth, typically, objectivity is evaluated based on the concordance rate of judgment results by multiple endoscopists, but in this study, as an alternative approach, a single endoscopist assessed the endoscopic findings, and objectivity was achieved using ImageJ software. Further validation of this method will be necessary in the future. Fifth, Fifth, due to the lack of consensus among pathologists regarding dysplasia, the criteria used in this study are still subject to debate, and they cannot be immediately generalized. Further investigation is required in the future.

In conclusion, SSLs with cancerous components including intramucosal carcinoma in Japan can be detected with high sensitivity when the lesion diameter is ≥10 mm and some reddening is present. Furthermore, visible vessels observed using magnifying NBI can be objective indicators of the diagnosis of SSLs with cancerous components with a high degree of accuracy. Precancerous neoplasia associated with SSLs may be easily identified with magnifying NBI because it can detect areas of visible vessels, as opposed to invisible vessel areas, of SSLs.

## CONFLICT OF INTEREST STATEMENT

None.

## Supporting information

Figure S1 Endoscopic findings of group A sessile serrated lesions (SSLs) with reddening. (Left): The yellow dotted line indicates the reddening of SSLs without cancerous components. (Middle): Narrow‐band imaging (NBI) findings of the reddening area (distant view). (Right): Magnifying NBI shows a lacy vessel pattern, indicating a noncancerous vascular pattern.

## References

[1] Leggett B , Whitehall V . Role of the serrated pathway in colorectal cancer pathogenesis. Gastroenterology 2010; 138: 2088–2100. 20420948 10.1053/j.gastro.2009.12.066

[2] Crockett SD , Nagtegaal ID . Terminology, molecular features, epidemiology, and management of serrated colorectal neoplasia. Gastroenterology 2019; 157: 949–966.e4. 31323292 10.1053/j.gastro.2019.06.041

[3] Snover DC , Ahnen DJ , Burt RW , Odze RD . Serrated polyps of the colon and rectum and serrated polyposis. In: WHO Classification of Tumours of the Digestive System, 4th edn, WHO Classification of Tumours Editorial Board, Lyon, France: IARC, 2010; 160–165.

[4] Vennelaganti S , Cuatrecasas M , Vennalaganti P *et al*. Interobserver agreement among pathologists in the differentiation of sessile serrated from hyperplastic polyps. Gastroenterology 2021; 160: 452–454. 32950521 10.1053/j.gastro.2020.09.015

[5] Pai RK , Mäkinen MJ , Rosty C . Colorectal serrated lesions and polyps. In: WHO Classification of Tumours of the Digestive System, 5th edn, WHO Classification of Tumours Editorial Board, Lyon, France: IARC, 2019; 163–169.

[6] Hashiguchi Y , Muro K , Saito Y *et al*. Japanese Society for Cancer of the Colon and Rectum (JSCCR) guidelines 2019 for the treatment of colorectal cancer. Int J Clin Oncol 2020; 25: 1–42. 31203527 10.1007/s10147-019-01485-z PMC6946738

[7] Nagtegaal ID , Arends MJ , Salto‐Tellez M . Colorectal adenocarcinoma. In: WHO Classification of Tumours of the Digestive System, 5th edn, WHO Classification of Tumours Editorial Board, Lyon, France: IARC, 2019; 177–187.

[8] Liu C , Walker NI , Leggett BA *et al*. Sessile serrated adenomas with dysplasia: Morphological patterns and correlations with MLH1 immunohistochemistry. Modern Pathol 2017; 30: 1728–1738. 10.1038/modpathol.2017.92 PMC5719122 28752838

[9] Cenal O , Gibson J , Odze R *et al*. Clinicopathologic and outcome study of sessile serrated adenomas/polyps with serrated versus intestinal dysplasia. Mordern Pathol 2018; 31: 633–642. 10.1038/modpathol.2017.169 29271414

[10] Chino A , Osumi H , Kishihara T *et al*. Advantages of magnifying narrow‐band imaging for diagnosing colorectal cancer coexisting with sessile serrated adenoma/polyp. Dig Endosc 2016; 28: 53–59. 26864801 10.1111/den.12631

[11] Sano Y , Tanaka S , Kudo SE *et al*. Narrow‐band imaging (NBI) magnifying endoscopic classification of colorectal tumors proposed by the Japan NBI Expert Team. Dig Endosc 2016; 28: 526–533. 26927367 10.1111/den.12644

[12] Yamashina T , Takeuchi Y , Uedo N *et al*. Diagnostic features of sessile serrated adenoma/polyps on magnifying narrow band imaging: A prospective study of diagnostic accuracy. J Gastroenterol Hepatol 2015; 30: 117–123. 25088839 10.1111/jgh.12688

[13] Bettington M , Walker N , Rosty C *et al*. Clinicopathological and molecular features of sessile serrated adenomas with dysplasia or carcinoma. Gut 2017; 66: 97–106. 26475632 10.1136/gutjnl-2015-310456

[14] Hattem WAV , Shahidi N , Vosko S *et al*. Piecemeal cold snare polypectomy versus conventional endoscopic mucosal resection for large sessile serrated lesions: A retrospective comparison across two successive periods. Gut 2021; 70: 1691–1697. 33172927 10.1136/gutjnl-2020-321753

[15] Chino A , Nagahama H , Ishikawa H *et al*. Cancer emerging from the recurrence of sessile serrated adenoma/polyp resected endoscopically 5 years ago. Jpn J Clin Oncol 2016; 46: 89–95. 26538462 10.1093/jjco/hyv154

[16] Murakami T , Sakamoto N , Ritsuno H *et al*. Distinct endoscopic characteristics of sessile serrated adenoma/polyp with and without dysplasia/carcinoma. Gastrointest Endosc 2017; 85: 590–600. 27663716 10.1016/j.gie.2016.09.018

[17] Sano W , Hirata D , Teramoto A *et al*. Serrated polyps of the colon and rectum: Remove or not? World J Gastroenterol 2020; 26: 2276–2285. 32476792 10.3748/wjg.v26.i19.2276 PMC7243646

[18] Saiki H , Nishida T , Yamamoto M *et al*. Frequency of coexistent carcinoma in sessile serrated adenoma/polyps and traditional serrated adenomas removed by endoscopic resection. Endosc Int Open 2016; 4: E451–E458. 27092327 10.1055/s-0042-103239 PMC4831921

[19] Lash RH , Genta RM , Schuler CM . Sessile serrated adenomas: Prevalence of dysplasia and carcinoma in 2139 patients. J Clin Pathol 2010; 63: 681–686. 20547691 10.1136/jcp.2010.075507

[20] Chino A , Yamamoto N , Kato Y *et al*. The frequency of early colorectal cancer derived from sessile serrated adenoma/polyps among 1858 serrated polyps from a single institution. Int J Colorectal Dis 2016; 31: 343–349. 26510850 10.1007/s00384-015-2416-2

[21] O'Brien MJ , Winawer SJ , Zauber AG *et al*. The National Polyp Study. Patient and polyp characteristics associated with high‐grade dysplasia in colorectal adenomas. Gastroenterology 1990; 98: 371–379. 2403953

