# Supplementary figures and images for: Advantage of magnifying narrow‐band imaging for the diagnosis of colorectal neoplasia associated with sessile serrated lesions
Source: DEN Open. 2023 Dec 1;4(1):e315. doi: 10.1002/deo2.315 (PMC10690695; doi:10.1002/deo2.315)

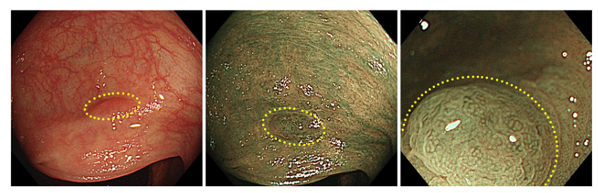

Supplement: Supplementary file 1 — Figure S1 Endoscopic findings of group A sessile serrated lesions (SSLs) with reddening. (Left): The yellow dotted line indicates the reddening of SSLs without cancerous components. (Middle): Narrow‐band imaging (NBI) findings of the reddening area (distant view). (Right): Magnifying NBI shows a lacy vessel pattern, indicating a noncancerous vascular pattern. [file DEO2-4-e315-s001.tiff]
